# The influence of generative artificial intelligence on creative cognition of design students: a chain mediation model of self-efficacy and anxiety

**DOI:** 10.3389/fpsyg.2024.1455015

**Published:** 2025-01-27

**Authors:** Younjung Hwang, Yi Wu

**Affiliations:** ^1^School of Design, Hunan University, Changsha, China; ^2^School of International Communication and Arts, Hainan University, Haikou, China

**Keywords:** generative AI, creative cognition, self-efficacy, anxiety, design education

## Abstract

**Introduction:**

This study investigated the role of generative Artificial Intelligence (AI) in enhancing the creative cognition of design students, examining the mediating effects of self-efficacy and anxiety reduction.

**Methods:**

A quantitative approach was employed, collecting data through online surveys from 121 design students at universities in southern China. The study utilized scales for AI knowledge and perception, self-efficacy, anxiety, and creative cognition, adapted from previous studies and evaluated on 5-point Likert scales. Data analysis was conducted using SPSS 24.0 for exploratory factor analysis and PROCESS v3.5 for mediation analysis.

**Results:**

The findings confirmed that AI positively impacted students’ innovative thinking (*β* = 0.610, *p* < 0.001). Self-efficacy (standardized *β* = 0.256, 95% CI [0.140, 0.418], *p* < 0.001) and anxiety reduction (standardized *β* = 0.093, 95% CI [0.018, 0.195], *p* < 0.05) positively mediated the relationship between generative AI and creative cognition. Additionally, a serial mediation effect through self-efficacy and anxiety reduction was observed (standardized *β* = 0.053, 95% CI [0.012, 0.114], *p* < 0.05).

**Discussion:**

Our empirical analysis demonstrates that AI positively affects design students’ innovative thinking, with self-efficacy and anxiety reduction serving as significant mediators. These findings provide valuable insights for educators and policymakers, suggesting that AI-integrated design curricula can significantly foster creative cognition, promote academic achievement, and enhance designer capabilities. Understanding AI’s impact on students’ creative processes is crucial for developing effective teaching strategies in today’s evolving educational landscape.

## 1 Introduction

In recent years, Artificial Intelligence (AI) has emerged as a transformative force across various domains, including education and creative industries. AI refers to computer systems capable of performing complex tasks traditionally associated with human cognition, such as reasoning, decision-making, and problem-solving (Russell and Norvig, 2020). Generative AI, a subset of AI, is designed to analyze patterns in large datasets and use these patterns to generate outcomes based on user requirements (Mandapuram et al., 2018; Liang et al., 2023). As this technology gains prominence in creative disciplines, its integration in design education sparks considerable debate among researchers and educators, with conflicting views on its impact on creativity and learning outcomes.

Some studies suggest that AI fosters learners’ creativity by providing new tools and perspectives. For instance, language models like ChatGPT and text-to-image models such as Midjourney, Stable Diffusion, and DALL-E are increasingly adopted to enhance human creative cognition – the process of generating innovative ideas (Voigt et al., 2023). These tools offer students rapid prototyping capabilities and access to vast databases of design inspiration, potentially expanding their creative horizons. However, other research indicates that AI might inhibit creativity by promoting over-reliance on machine-generated solutions (Elgammal et al., 2017). Critics argue that excessive dependence on AI tools could lead to a homogenization of design outputs and a decrease in original, human-driven creativity.

Furthermore, in design education, the integration of AI tools not only introduces new creative methods but also impacts the psychological dynamics of learning. Key factors such as self-efficacy and anxiety influence how students engage with AI and, consequently, their creative outcomes (Runco and Chand, 1995; Sifonis and Ward, 2022). Despite AI’s growing presence in educational settings, little is known about how these psychological mechanisms shape creative cognition and design education. This gap in knowledge underscores the importance of understanding the complex interrelationship between AI and creative cognition for both students and educators in the field of visual design.

This ongoing debate highlights the need to explore how the integration of AI in design education affects students’ creative processes and to identify the psychological mechanisms through which AI influences creative cognition. Specifically, examining the roles of self-efficacy and anxiety in mediating the relationship between AI use and creative output is crucial for understanding how AI can best support creative learning. To address these gaps in knowledge, this study aimed to explore these effects, focusing on the roles of self-efficacy and anxiety, and offering insights into how AI can optimally support creative learning in design education.

### 1.1 Self-efficacy and creative cognition

The concept of self-efficacy, which refers to the individual’s belief in their ability to successfully perform tasks, plays a crucial role in promoting creative cognition. Bandura (1977) introduced the concept of self-efficacy and its impact on human behavior, while Beghetto (2006) specifically explored its relationship with creative performance in educational settings. Beghetto’s study of 1,322 middle and secondary school students found significant positive relationships between creative self-efficacy and several factors related to creative performance, including mastery orientation (*β* = 0.30, *p* < 0.001), performance-approach orientation (*β* = 0.12, *p* < 0.001), and teacher feedback on creative ability (*β* = 0.32, *p* < 0.001). This finding suggested that students who believe in their creative abilities are more likely to engage in creative endeavors and produce innovative outcomes.

Building on this foundation, McGuire et al. (2024) examined the effects of AI collaboration on creative self-efficacy and creativity in poetry writing. Their study found that participants who co-created poems with AI reported significantly higher levels of creative self-efficacy compared to those who merely edited AI-generated poems (*M* = 4.62 vs. *M* = 3.74, *p* = 0.003). This increased self-efficacy translated into higher expert evaluations of creativity for the co-creator group compared to the editor group (*M* = 14.70 vs. *M* = 12.53, *p* = 0.026). Mediation analysis revealed that creative self-efficacy significantly mediated the relationship between co-creation with AI and expert evaluations of creativity (indirect effect, *β = 0*.78, *SE* = 0.39, 95% CI [0.16, 1.68]). These findings suggest that when properly designed to foster co-creation, AI tools can enhance users’ creative self-efficacy, leading to improved creative outcomes. However, a limitation of this research is that it does not address the long-term impact of co-creating versus editing with AI, as the study focused on a single creative task rather than examining effects over an extended period of time.

### 1.2 Anxiety and creative cognition

Another important factor in the creative process is anxiety, a negative emotion, which is characterized by tension, physical ailments, and worrisome thoughts (Spielberger, 1972). Creative anxiety is particularly common among designers and design students (Daker et al., 2020; Wang and Jia, 2021). Research has shown that reducing anxiety can enhance students’ creative cognition by freeing up cognitive resources typically consumed by stress, thus facilitating the development of innovative ideas. In a meta-analysis of 76 experimental studies, Byron et al. (2010) found that low-anxiety individuals showed significantly increased creative performance when exposed to stressors compared to high-anxiety individuals (*d* = 0.39, 95% CI [0.19, 0.62] vs. *d* = −0.12, 95% CI [−0.33, 0.09], respectively). This suggests that interventions aimed at reducing anxiety could potentially improve creative outcomes in design education.

According to recent research, the relationship between anxiety and self-efficacy in creative design contexts appears to be influenced by AI-generated content (AIGC) tools. Based on Li’s (2024) study of 404 design students and professionals, the relationship between anxiety, self-efficacy, and AI in creative design contexts reveals significant insights. While AI tools did not directly reduce anxiety, they indirectly influenced it through other factors. The study found that performance expectancy (*β* = 0.57, *p* < 0.001) and social influence (*β* = 0.24, *p* < 0.001) positively impacted designers’ intention to use AI tools. This suggests that as designers perceive AI tools as enhancing their performance and gain social support, their self-efficacy may increase, potentially leading to reduced anxiety about creative tasks. However, this study did not directly measure changes in anxiety or self-efficacy levels over time, limiting our understanding of AI’s long-term psychological impact on designers.

### 1.3 Research aims and hypotheses

Considering these various factors, given the complex interrelationships and gaps in current research, this study aimed to explore the connections among self-efficacy, anxiety, AI design knowledge, and creative cognition in the context of design education. The research sought to address how AI tools in design education affect students’ creative cognition, the extent to which self-efficacy mediates this relationship, and how anxiety reduction contributes to the enhancement of creative cognition through AI tools. Additionally, the study examined the potential sequential mediating effect of self-efficacy and anxiety reduction in the relationship between AI use and creative cognition.

The researchers hypothesized that AI tools would initially boost students’ self-efficacy by providing rapid prototyping and ideation support. This increased self-efficacy was expected to lead to reduced anxiety about the creative process as students felt more capable of tackling design challenges. Consequently, the combination of enhanced self-efficacy and reduced anxiety was hypothesized to facilitate improved creative cognition, enabling students to generate more innovative and effective design solutions.

Through this investigation, the study aimed to contribute empirical evidence to support the connections between AI tools and creative cognition, specifically examining how self-efficacy and anxiety reduction mediate this relationship in the context of design education. By clarifying the mechanisms through which AI influences creative cognition, the research sought to inform the development of more effective AI-integrated design curricula and offer valuable insights for educators striving to harness AI’s potential while addressing its possible drawbacks.

## 2 Literature review

### 2.1 Generative AI for enhancing students’ creative cognition

Creative cognition encompasses the cognitive processes involved in generating novel and innovative ideas, engaging in divergent thinking, and exhibiting creativity that goes beyond routine problem-solving. This creative thinking is characterized by flexibility, originality, and the ability to make unexpected connections, distinguishing it from more conventional, linear thought processes used in everyday tasks (Smith et al., 1995; Ahmad Abraham and Windmann, 2007; Runco and Acar, 2012). It also includes metacognitive elements, which refer to higher-order thinking processes that allow individuals to reflect on, monitor, and control their cognitive activities during creative work (Amabile, 1983; Hargrove and Nietfeld, 2015). These metacognitive processes involve strategic planning, evaluating progress, and adjusting approaches as needed. For instance, a designer generating multiple ideas is engaging in cognitive activity, while assessing the effectiveness of their ideation strategy, altering their approach if necessary, and reflecting on how well they are meeting project goals represents metacognitive activity (Jaušovec, 1994; Kaufman and Beghetto, 2013). The interplay between cognitive and metacognitive processes in creative work is complex and dynamic. While problem-solving can involve both cognitive and metacognitive elements, the distinction lies in the level of conscious reflection and control. Routine problem-solving may rely more heavily on cognitive processes, while novel or complex problem-solving often requires more explicit metacognitive engagement (Sternberg and Lubart, 1996; Mumford et al., 2012).

Furthermore, as our understanding of human creativity continues to evolve, advancements in AI have introduced new tools that may complement and enhance cognitive processes. Generative AI, in particular, has garnered significant attention for its potential to support and expand creative cognition. Designed to analyze patterns in large datasets and generate outcomes based on user requirements (Mandapuram et al., 2018; Liang et al., 2023), generative AI has made significant strides in various domains. Large language models (LLMs) like ChatGPT have revolutionized text generation and comprehension (Tholander and Jonsson, 2023; Lanzi and Loiacono, 2023; Tan and Luhrs, 2024), while image generation models such as Midjourney, Stable Diffusion, and DALL-E 2 have transformed visual creative processes (Paananen et al., 2023; Hanafy, 2023; Yüksel and Börklü, 2023). As these technologies continue to evolve, their applications have expanded far beyond their original domains, sparking growing interest in fields such as education (Sadek, 2023; Slimi, 2023).

In educational settings, the integration of generative AI has shown promise in enhancing students’ creative cognition. These AI systems serve as collaborative tools, bridging the gap between data scientists and non-experts, and enabling individuals without prior knowledge of data analysis and machine learning to understand and utilize complex concepts through conversation (Li, 2023). By engaging in dialogue with students, AI made difficult topics more accessible by providing explanations tailored to individual understanding levels.

This approach offers opportunities for students from diverse backgrounds and knowledge levels to grasp and apply complex concepts more easily. For instance, a student struggling with a particular machine learning algorithm could ask ChatGPT to explain it in simpler terms or relate it to familiar concepts (Islam et al., 2023), potentially enhancing students’ creativity by making it easier for learners from diverse backgrounds to grasp and apply concepts in data science and machine learning.

This capability of generative AI not only facilitates learning but also promotes creativity by making advanced concepts more approachable and manageable, thus contributing to the development of students’ innovative thinking skills.

### 2.2 Self-efficacy as a mediator

Self-efficacy, defined as an individual’s belief in their ability to successfully perform tasks, has been shown to significantly impact creative activities and outcomes (Bandura, 1977; Maddux, 2013; Rahayuningsih et al., 2022; Yu et al., 2023; Bozdoğan, 2023; Raihan and Uddin, 2023). In the context of education, self-efficacy plays a crucial role in the development of undergraduate students’ creative cognitive abilities (Sri et al., 2019; Beghetto, 2006; Qian et al., 2023; Liang et al., 2023). For instance, Beghetto (2006) found a significant positive correlation between creative self-efficacy and self-reported creative performance among middle and secondary school students (*r* = 0.572, *p* < 0.001). Similarly, Qian et al. (2023) demonstrated that self-directed learning positively influences creativity in healthcare undergraduates through the mediating effects of openness to challenge and diversity, as well as creative self-efficacy (indirect effect = 0.324, 95% CI [0.165, 0.543]).

Building on this understanding, recent research has explored the potential influence of AI on students’ self-efficacy and motivation. Jia and Tu (2024) investigated the impact of AI capabilities on college students’ self-efficacy, finding a significant positive relationship (*β* = 0.546, *p* < 0.001). Expanding on this, Yilmaz and Yilmaz (2023) examined the effects of AI-based tools on programming self-efficacy, revealing that students using AI tools scored significantly higher in programming self-efficacy compared to the control group (*F*(1, 42) = 15.144, *p* < 0.001).

Furthermore, Wang and Chuang (2023) explored the effects of higher education institutes’ AI capability on students’ self-efficacy, creativity, and learning performance. Their study indicated that AI capability significantly affects students’ self-efficacy (*β* = 0.515, *p* < 0.001) and creativity (*β* = 0.533, *p* < 0.001), which in turn positively influence learning performance.

This transformative impact of AI extends to specific domains within education, such as design-related fields, where the enhancement of self-efficacy through AI-assisted learning significantly influences students’ creative processes. Research has shown that increased confidence in design, partly facilitated by AI tools, promotes students’ independent and divergent thinking, enabling them to express and experiment with their ideas more freely. In this context, Rao et al. (2020) conducted a study with 150 design students, finding that those who reported higher levels of confidence in their design abilities were 30% more likely to propose innovative solutions to given design problems. The researchers attributed this increased confidence partly to the use of AI-assisted design tools. Similarly, Liu et al. (2023) observed in their longitudinal study of 200 undergraduate design students that increased confidence, partly attributed to AI-assisted learning tools, correlated with a 25% increase in the originality of design projects over a two-year period.

Furthermore, this willingness to embrace and explore novel concepts and perspectives, fostered by AI-enhanced learning environments, enhances flexible problem-solving abilities, which in turn fosters the development of creative cognitive processes. Kusmiyati (2022) demonstrated, in an experimental study with 100 graphic design students that those who received AI-enhanced feedback on their work showed a 40% improvement in their ability to generate multiple design solutions for a single problem. Giancola et al. (2022) further supported this finding in their research involving 180 architecture students, where AI-assisted design tools led to a 35% increase in the students’ willingness to explore unconventional design approaches. As a result, AI-assisted learning boosts students’ confidence in their design skills, encouraging them to attempt novel approaches rather than adhering to conventional methods. This confidence can lead to more innovative and creative outcomes in design education.

Beyond specific domains, AI plays a crucial role in enhancing the overall learning experience by supporting learners’ self-regulation abilities. Self-regulation in learning refers to the process by which learners actively manage their thoughts, behaviors, and emotions to successfully navigate their learning experiences (Zimmerman, 2002; Schunk and Zimmerman, 2011). These abilities are crucial for learners to effectively set and achieve goals, assess progress, and make necessary adjustments to enhance performance (Lemos, 1999). For instance, the Adaptive Immediate Feedback (AIF) system offers real-time shaping feedback to students during programming tasks, enhancing their aesthetic and critical skills (Hooda et al., 2022). This type of AI-driven support provides learners with personalized guidance and assessment, potentially improving their ability to self-regulate their learning.

In this context, AI systems have the potential to provide learners with personalized guidance and assessment. This support can enhance the learning process, which may indirectly contribute to the development of creative cognitive abilities. However, more research is needed to establish a clear causal relationship between AI-enhanced self-efficacy and improved creative cognition.

### 2.3 Anxiety as a mediator

Anxiety, an emotion characterized by tension, worrisome thoughts, and physical changes, plays a significant role in creative processes. Barlow and Durand (2015) define anxiety as “a negative mood state characterized by symptoms of physical tension and apprehension about the future” (p. 123). In the context of creativity, anxiety can significantly affect performance and cognitive processes. This impact is particularly evident in creative anxiety, which is common among designers and students.

Creative anxiety often stems from psychological stress and low self-esteem (Rudra et al., 2012; Garner, 2015). Various factors can trigger this anxiety, including the ambiguity of creative tasks, high expectations, time pressure, or fear of criticism (Vlăduțu et al., 2019). Interestingly, research shows that anxiety levels can influence creative performance differently. For instance, Wågan et al. (2021) find that individuals with high anxiety levels experience a decline in creative performance under acute stress, while those with low anxiety levels enhance their creative cognitive abilities in specific tasks. However, Guo et al. (2024) and Lee et al. (2024) demonstrate that, regardless of initial anxiety levels, acute stress generally impairs creativity and reduces cognitive flexibility. These findings highlight the need for effective interventions to manage anxiety in creative contexts.

AI platforms offer personalized learning experiences that may help alleviate academic anxiety and minimize stress associated with meeting academic expectations. Toribio (2023) surveys 500 high school students using AI-powered adaptive learning software and found a 30% reduction in self-reported academic stress levels. Furthermore, Wang et al. (2022) conduct a meta-analysis of 20 studies on AI-assisted personalized learning, revealing a moderate positive effect on reducing academic anxiety (Cohen’s *d* = 0.48). These findings are supported by Fulmer et al.’s (2018) longitudinal study of 300 college students, which notes a significant decrease in cortisol levels over a semester of using AI-driven feedback systems.

Building on these insights, the stress reduction facilitated by AI tools could have particular significance for creative cognition. Using fMRI technology, Guo et al. (2024) demonstrate that acute stress impairs activity in brain regions associated with creativity. Similarly, Lee et al. (2024) observe a 25% decrease in performance on divergent thinking tests among participants exposed to stressful tasks compared to a relaxed control group. Consequently, AI platforms further enhance these benefits through their bidirectional communication systems, potentially alleviating psychological tension (Parekh et al., 2023; Sadeh-Sharvit et al., 2023). For example, ChatGPT’s ability to explain concepts, answer follow-up questions, and maintain continuous interaction provides a supportive learning environment (Du and Alm, 2024). Additionally, the ease with which users can manipulate images using generative AI prompts may help reduce students’ sense of burden and tension regarding creativity. This effect is evidenced by Rasouli et al.'s (2022) survey of 300 design students using AI-assisted creative tools, where 78% reported feeling less pressured and more confident in their creative abilities. This increased confidence and reduced anxiety can have far-reaching effects on creative cognition.

Moreover, the optimistic mindset fostered by AI interaction can liberate individuals from task constraints, provide new insights, and enable more abstract thinking. Plucker (2021) suggests that this expanded cognitive space enhances creative cognition by facilitating the combination of different ideas. Consequently, the seamless interaction with generative AI for tasks such as image generation could counteract the negative impacts of creative challenges and alleviate unnecessary tension, potentially contributing to the enhancement of students’ creative cognition.

### 2.4 Research hypotheses

Based on an assessment of findings from the aforementioned research works, the following hypotheses were proposed:

*Hypothesis 1 (H1): The use of generative AI would improve students’ creative cognitive skills, particularly in terms of creative thinking and ideation.*

*Hypothesis 2 (H2): The use of generative AI would increase students’ self-efficacy and decrease their anxiety.*

*Hypothesis 3 (H3): The increase in students’ self-efficacy due to the use of generative AI would mediate the improvement of creative cognitive ability.*

*Hypothesis 4 (H4): Reducing students’ task-related anxiety due to the use of generative AI would mediate the improvement of creative cognitive ability.*

*Hypothesis 5 (H5): The use of generative AI would increase students’ self-efficacy and decrease their anxiety, and these two factors would sequentially mediate the enhancement of creative cognitive abilities.*

**Figure 1:**
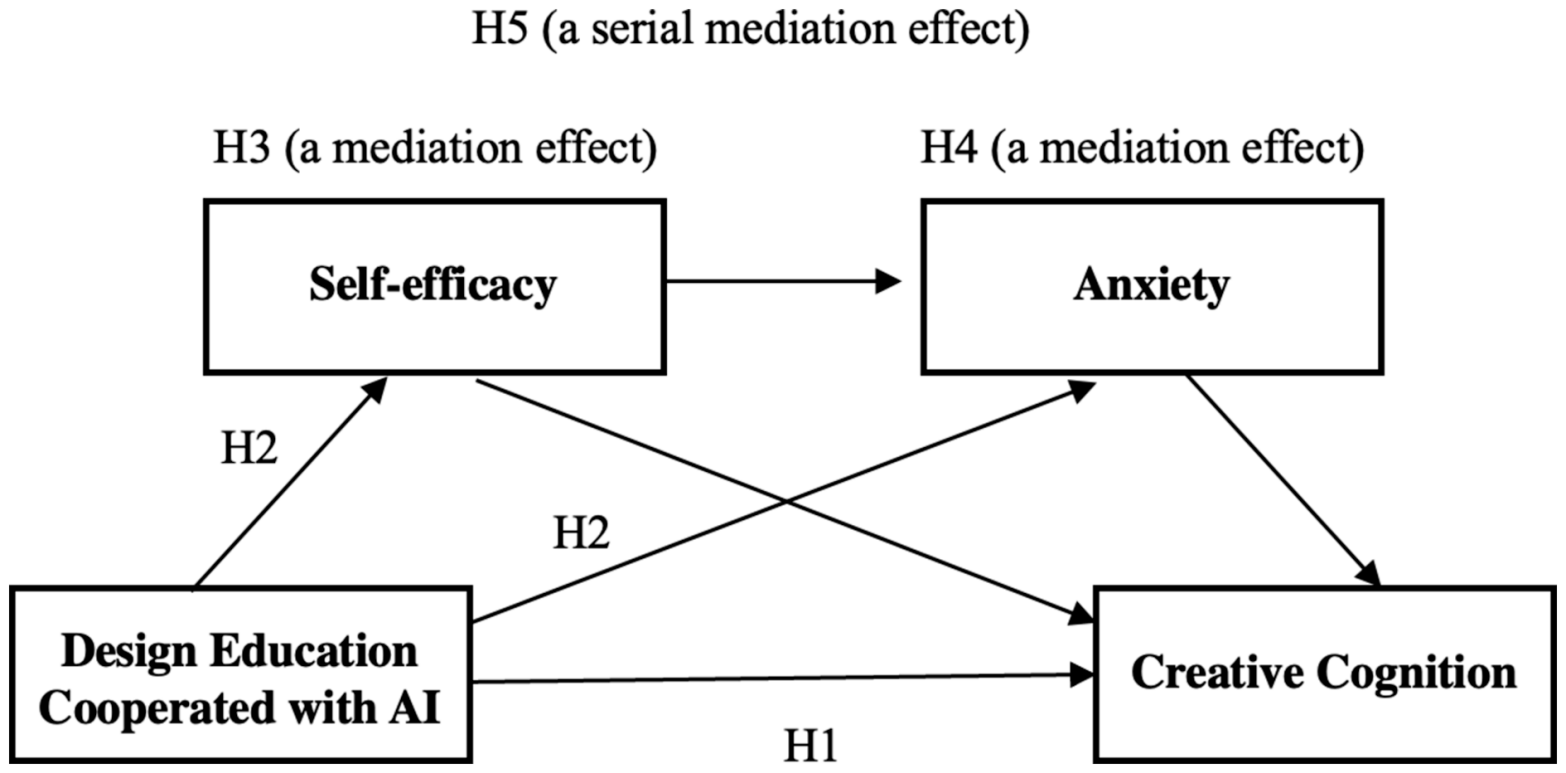
Hypothesized research model.

The hypothesized research model, which visually represents these proposed relationships, is presented in Figure 1.

## 3 Methodology

### 3.1 Sample and procedure

To address the research questions and test the proposed hypotheses, this study employed a quantitative approach using online surveys. Data collection was conducted for 1 month in February 2024, targeting university students majoring in design at various institutions in southern China. This sampling strategy was chosen to ensure a diverse representation of design disciplines and to capture the experiences of students who engaged with generative AI tools in their academic and creative work. For image-generative AI, students generally utilized Midjourney (Version 6.0, developed by Midjourney Inc.) and DALL-E (Version 3.0, developed by OpenAI).

To achieve this, the study gathered responses from students across various design specializations, including visual design, industrial design, and other related fields. This diverse sample allowed for a comprehensive examination of the impact of generative AI on creative cognitive abilities across different design domains. For practical considerations, the online survey method was selected for its efficiency in reaching a wide range of participants and its ability to standardize data collection procedures.

Consequently, a total of 121 completed questionnaires were collected and used for data analysis. In terms of sample characteristics, the demographic characteristics of the respondents provided a balanced representation of the target population. Out of the 121 respondents, 45 were male (37.2%) and 76 were female (62.8%), reflecting a gender distribution that was consistent with typical enrollment patterns in design programs. The academic year distribution of the participants was as follows: 68 were first-year students (56.2%), 6 were second-year students (5.0%), 15 were third-year students (12.4%), and 32 were in their fourth year or above (26.4%) (see Table 1).

**Table 1:** Participant characteristics.

Characteristic	Variables	*n*	%
Gender	Male	45	37.2
	Female	76	62.8
Academic year	First year	68	56.2
	Second year	6	5.0
	Third year	15	12.4
	Fourth year or above	32	26.4
Design major	Visual design	40	33.1
	Industrial design	35	28.9
	UX/UI design	25	20.7
	Fashion design	21	17.3

*N = 121.*

Notably, this diverse sample in terms of gender and academic progression allowed for a nuanced analysis of how the use of generative AI might impact students at different stages of their design education. The inclusion of students from various academic years provided an opportunity to examine whether the effects of generative AI on creative cognition, self-efficacy, and anxiety levels varied based on students’ level of experience and expertise in their chosen design field.

### 3.2 Measurements

The questionnaire for this study comprises four key sections: knowledge and perception of AI (10 items), self-efficacy (5 items), anxiety reduction (5 items), and creative cognition (5 items). For example, the original item “I feel I can solve difficult aspects of design tasks” was adjusted to “I feel I can solve difficult aspects of design tasks using generative AI.” Prior to the main survey, participants received a clear definition and examples of generative AI in the context of design education to ensure a common understanding of the term.

All items were evaluated on a 5-point Likert scale ranging from 1 (strongly disagree) to 5 (strongly agree). The Artificial Intelligence and Design (AID) scale developed for this study was adapted from various relevant studies. Starting with the first component, the knowledge and perception of AI measure is based on the AI and Design scale, which incorporates elements from AI and learning scales by Kim and Lee (2022) and Chai et al. (2024). Sample items include: “I have general knowledge about the use of generative AI,” and “I can express the design I want through generative AI.” Moving to the second component, the Self-Efficacy measure is derived from AI self-efficacy scales developed by Xia et al. (2022) and Wang and Chuang (2023), and adapted for the AI and Design context. Sample items include: “I feel I can solve difficult aspects of design tasks using AI,” and “I have gained confidence in design tasks by using AI.” For the third component, to measure anxiety reduction, items from the AID scale are modified from the Creativity Anxiety Scale (CAS) developed by Daker et al. (2020). Sample items include: “Using AI has reduced tension in design tasks,” and “Using AI has decreased fear of a blank screen.” For the fourth component, the creative cognition measure within the AID scale is adapted from the Creative Cognition Scale developed by Miller (2014). Sample items include: “I have been able to derive creative designs by connecting different types of ideas using AI,” and “I have been able to discover solutions to my design problems from different perspectives through AI.”

Taken together, this comprehensive questionnaire structure, comprising the AID scale along with subscales for self-efficacy, anxiety reduction, creative cognition, and knowledge and perception of AI, allows for the collection of detailed insights into participants’ experiences with generative AI in design education.

### 3.3 Statistical processing

This study employed various statistical analyses to examine our research hypotheses. Using SPSS 24.0, we first conducted an exploratory factor analysis (EFA) to examine the structural validity of our AID scale. This scale was designed to measure four distinct constructs (AI knowledge and perception, self-efficacy, anxiety reduction, and creative cognition) within a single instrument. We included all items in a single EFA to assess the construct validity of our measure, check for any unexpected cross-loadings between the scale’s components, and verify that the empirical factor structure matched our theoretical expectations across all components simultaneously. For the EFA, we employed principal component analysis with varimax rotation, as the components within our scale were conceptually distinct yet part of a single measure.

Prior to the main analysis, to determine the suitability of our data for factor analysis, we conducted KMO and Bartlett’s tests. The KMO test examined the correlation and partial correlation between variables, with values above 0.8 suggesting that the data was suitable for factor analysis. The Bartlett’s test of sphericity was used to determine whether each variable was independent, supporting our choice of varimax rotation within our scale.

To analyze the reliability of each component within our unified AID scale, we employed Cronbach’s alpha coefficient. In interpreting the results, values above 0.7 were considered acceptable, and values above 0.8 indicated good reliability.

Following the EFA and reliability analysis, we applied the mediation analysis approach proposed by Baron and Kenny (1986) to analyze the relationships among design education using generative AI, self-efficacy, anxiety, and creative cognition, sequentially verifying both direct and indirect (mediating) effects.

We applied four regression models. Model 1 examined the effect of generative AI in design education on creative cognition. Model 2 assessed how generative AI in design education influences self-efficacy. Model 3 analyzed the effects of generative AI in design education and self-efficacy on anxiety. Model 4 examined the overall relationships among generative AI in design education, self-efficacy, anxiety, and creative cognition. Each model was linked directly to our research hypotheses, with Hypotheses 1 through 5 (H1–H5) tested through various combinations of these models.

Additionally, to enhance the robustness of our mediation analysis, we applied the bootstrapping method using PROCESS version 3.5 (Hayes, 2017). This approach was chosen for its robustness to non-normality in the sampling distribution of indirect effects, higher statistical power in complex models, and ability to estimate confidence intervals for indirect effects. We generated 95% bias-corrected confidence intervals through 5,000 bootstrap resamples, considering the mediating effect statistically significant if the confidence interval did not include zero.

This comprehensive statistical approach, combining exploratory factor analysis, reliability testing, mediation analysis, and bootstrapping, allowed us to thoroughly examine our research hypotheses and provide robust insights into the relationships among generative AI use, self-efficacy, anxiety, and creative cognition in the context of design education, as measured by our unified AID scale.

## 4 Results

### 4.1 Exploratory factor analysis

To examine the structural validity of our measurement tool, the AID scale, we conducted an exploratory factor analysis (EFA). This scale comprised of four components: knowledge and perception of AI, self-efficacy, anxiety reduction, and creative cognition. Prior to the main analysis, we performed KMO and Bartlett’s tests to determine the suitability of our data for factor analysis.

Upon initial examination, the results of these preliminary tests were encouraging. The KMO value was 0.916, well above the recommended threshold of 0.8, indicating that our data was very suitable for factor analysis. Additionally, the Bartlett’s test of sphericity yielded a significance level less than 0.001, rejecting the null hypothesis of variable independence and further confirming the appropriateness of factor analysis for our data.

Given these positive indicators, we proceeded with the EFA using principal component analysis with varimax rotation, an orthogonal rotation method. This approach was chosen because our scale was conceptually distinct and not expected to be correlated, an assumption supported by the results of Bartlett’s test of sphericity. Based on the theoretical framework of our study, we extracted four principal component variables.

Our initial analysis revealed that two items had factor loadings greater than 0.40 on multiple factors, exceeding our predetermined threshold for acceptable cross-loadings. Specifically, one item from the anxiety reduction component (“Using AI has decreased fear of a blank screen”) and one item from the self-efficacy component (“I have gained confidence in design tasks by using AI”) showed these high cross-loadings. To maintain the clarity and distinctiveness of our factors, we made the decision to remove these items from the analysis.

Following the removal of these two items, we conducted a second rotation with the remaining items. This adjustment resulted in a clean four-factor structure that aligned well with our theoretical constructs. These factors were labeled as follows: design education cooperated with AI, self-efficacy, anxiety, and creative cognition.

As a result of this process, this rigorous approach to factor analysis allowed us to confirm the structural validity of our AID scale while also refining it to ensure clear and distinct factors. The resulting four-factor structure provided a solid foundation for our subsequent analyses, aligning closely with the theoretical framework underpinning our study of generative AI in design education.

The rotated factor loadings using varimax rotation are presented in Table 2. The distribution of factor items remained consistent with the theoretical distribution. All reported factor loadings are greater than 0.5, indicating good construct validity for the scale items.

**Table 2:** Rotated component matrix.

Items	Factor 1 (design education)	Factor 2 (anxiety)	Factor 3 (self-efficacy)	Factor 4 (creative cognition)
DE9	**0.783**	0.124	0.231	0.156
DE5	**0.772**	0.098	0.187	0.143
DE1	**0.759**	0.112	0.201	0.178
DE2	**0.725**	0.089	0.167	0.132
DE3	**0.721**	0.103	0.189	0.145
DE10	**0.685**	0.078	0.154	0.121
DE6	**0.667**	0.087	0.176	0.134
DE8	**0.638**	0.092	0.165	0.128
DE4	**0.612**	0.076	0.143	0.112
AX3	0.098	**0.805**	0.187	0.165
AX4	0.087	**0.739**	0.176	0.154
AX1	0.092	**0.721**	0.165	0.143
AX2	0.076	**0.638**	0.143	0.132
SE3	0.201	0.176	**0.803**	0.189
SE5	0.187	0.165	**0.775**	0.176
SE1	0.189	0.154	**0.769**	0.165
SE2	0.167	0.143	**0.748**	0.154
SE4	0.154	0.132	**0.729**	0.143
CC4	0.156	0.145	0.189	**0.818**
CC3	0.143	0.134	0.176	**0.792**
CC5	0.132	0.128	0.165	**0.741**
CC1	0.128	0.121	0.154	**0.733**
CC2	0.121	0.112	0.143	**0.712**

Factor loadings > 0.40 are in boldface.

DE, design education cooperated with AI; AX, anxiety; SE, self-efficacy; CC, creative cognition.

Extraction Method: Principal Component Analysis.

Rotation Method: Varimax with Kaiser Normalization. Rotation converged in 6 iterations.

### 4.2 Reliability of the AID scale

Our analysis revealed high internal consistency for our comprehensive AID scale as a whole, as well as for its four components. The overall scale demonstrated excellent reliability (*α* = 0.945, 23 items). The individual components also showed strong internal consistency: design education cooperated with AI (*α* = 0.911, 9 items), anxiety reduction (*α* = 0.877, 4 items), self-efficacy (*α* = 0.914, 5 items), and creative cognition (*α* = 0.923, 5 items). All components demonstrated good reliability with Cronbach’s alpha values well above the 0.8 threshold, suggesting that the items in each component were effectively measuring the same underlying construct.

### 4.3 Descriptive statistics and correlation of variables

This study conducted descriptive statistics and correlation analysis on the main variables. The mean scores for design education cooperated with AI, anxiety, self-efficacy, and creative cognition were 3.571, 3.647, 3.455, and 3.628, respectively. These scores, all falling between 3.4 and 3.7, suggest that students have moderate levels of anxiety, self-efficacy, and creative cognition in the context of AI-integrated design education. Correlation analysis revealed that design education cooperated with AI correlated significantly with creative cognition (*r* = 0.606, *p* < 0.001), anxiety (*r* = 0.632, *p* < 0.001), and self-efficacy (*r* = 0.703, *p* < 0.001) (see Table 3).

**Table 3:** Description statistics and correlation of each variable.

	M	SD	1	2	3	4	5	6
1. Academic year	–	–	–					
2. Gender	–	–	0.060	–				
3. Design education cooperated with AI	3.571	0.688	0.113	045	–			
4. Anxiety	3.647	0.674	0.084	−0.029	0.584***	–		
5. Self-efficacy	3.455	0.756	0.043	−0.049	0.526***	0.599***	–	
6. Creative cognition	3.628		0.056	−0.035	0.606***	0.632***	0.703***	–

*N = 121. M and SD are used to represent mean and standard deviation, respectively.*

*p < 0.05. ** *p* < 0.01. *** *p* < 0.001.

### 4.4 Regression analysis of design education cooperated with AI, anxiety, self-efficacy, and creative cognition

This study employed linear regression analysis to examine the relationships among design education cooperated with AI, anxiety reduction, self-efficacy, and creative cognition. Four regression models were established based on the hypothesized relationships between these variables. The following research results were obtained:

The results of all four models were significant (*p* < 0.001). Model 1 showed that design education cooperated with AI had a significant positive impact on creative cognition. Model 2 revealed a significant positive relationship between design education cooperated with AI and self-efficacy. In Model 3, both design education cooperated with AI and self-efficacy demonstrated significant positive impacts on anxiety. Model 4 examined the interrelationships among all variables, confirming the positive impacts of design education cooperated with AI, self-efficacy, and anxiety on creative cognition. Detailed statistical results for all models are presented in Table 4.

**Table 4:** Regression analysis of design education cooperated with AI, anxiety, self-efficacy, and creative cognition.

			Fits the index			Regression coefficients		
Model	DV	IV	*R*	*R2*	*F*	*β*	*t*	*p*
Model 1	Creative cognition	Gender	0.609	0.371	23.02***	−0.009	−0.12	0.903
		Academic year				−0.006	−0.84	0.402
		Design education cooperated with AI				0.610	8.26	
								<0.001
Model 2	Self-efficacy	Gender	0.531	0.282	15.35***	−0.013	0.87	0.386
		Academic year				−0.072	0.36	0.719
		Design education cooperated with AI				0.531	6.73	
								<0.001
Model 3	Anxiety	Gender	0.678	0.459	24.65***	0.027	0.39	0.697
		Academic year				−0.028	--0.41	0.682
		Design education cooperated with AI				0.371	4.58	
								<0.001
		Self-efficacy				0.401	4.98	<0.001
Model 4	Creative cognition	Gender	0.755	0.600	34.51***	−0.008	−0.14	0.889
		Academic year				−0.017	−0.29	0.772
		Design education cooperated with AI				0.245	3.22	
								0.002
		Self-efficacy				0.438	5.71	<0.001
		Anxiety				0.227	2.83	0.005

DV, dependent variable; IV, independent variable.

*p < 0.05. **p < 0.01. ***p < 0.001.

### 4.5 Mediation effect test

The results of the mediation analysis supported the hypothesized indirect effects. The path from design education cooperated with AI to creative cognition through self-efficacy was significant. Similarly, the indirect path through anxiety was also significant. The serial mediation path from design education cooperated with AI to creative cognition through both self-efficacy and anxiety was significant as well. All confidence intervals for these indirect effects did not include zero, confirming their statistical significance. Detailed statistical results for the mediation analysis are presented in Table 5.

**Table 5:** Mediation effect test.

	95% CI				
Effect	*β* (standardized path coefficient)	*SE*	*LL*	*UL*	Effect size ratio (%)
Direct effect	0.267	0.083	0.103	0.431	39.91
Indirect effect 1	0.256	0.072	0.140	0.418	38.27
Indirect effect 2	0.093	0.045	0.018	0.195	13.90
Indirect effect 3	0.053	0.026	0.012	0.114	7.92
Total indirect effect	0.402	0.077	0.266	0.567	60.09
Total effect	0.669	0.081	0.510	0.829	100

CI, confidence interval; LL, lower limit; UL, upper limit.

Direct effect: The immediate effect of design education cooperated with AI on creative cognition.

Total indirect effect: The sum of all indirect effects (1, 2, and 3) of design education cooperated with AI on creative cognition through mediating variables.

Total effect: The sum of the direct effect and all indirect effects.

Indirect effect 1: Design education cooperated with AI → self-efficacy → creative cognition.

Indirect effect 2: Design education cooperated with AI → anxiety → creative cognition.

Indirect effect 3: Design education cooperated with AI → self-efficacy → anxiety → creative cognition.

**Figure 2:**
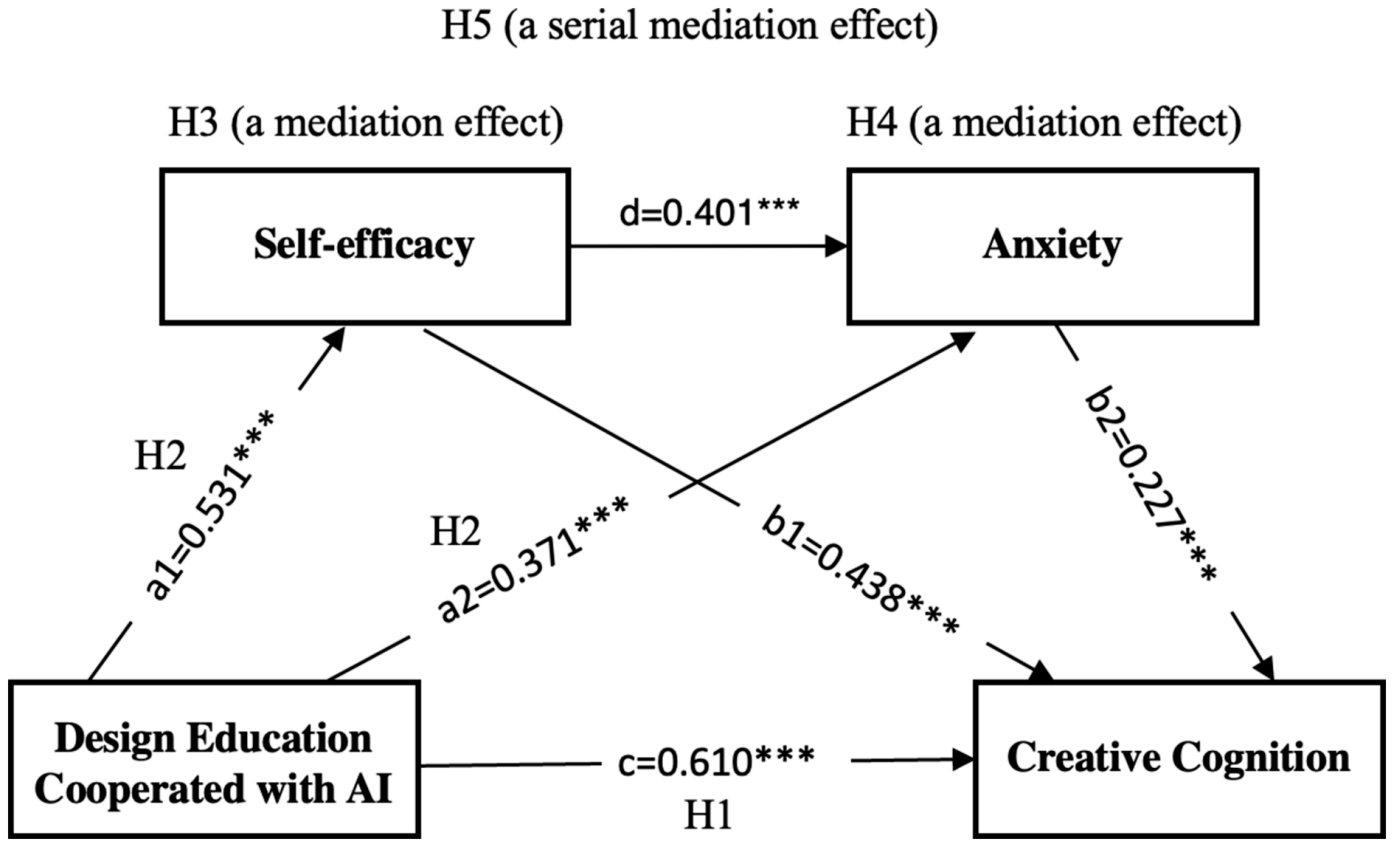
Relation model map.

Based on the findings of this study, Figure 2 illustrates the complex relationships between design education cooperated with AI, self-efficacy, anxiety, and creative cognition. The diagram reveals a series of direct and indirect effects that highlight the multifaceted impact of AI-integrated design education. Design education cooperated with AI demonstrates a strong positive direct effect on creative cognition (*β* = 0.610, *p* < 0.001), indicating that this educational approach significantly enhances students’ creative abilities. Additionally, the model shows two parallel mediation pathways: one through self-efficacy (H3) and another through anxiety (H4). The AI-integrated design education positively influences self-efficacy (*β* = 0.531, *p* < 0.001), which in turn has a positive effect on creative cognition (*β* = 0.438, *p* < 0.001). Conversely, it also impacts anxiety (*β* = 0.371, *p* < 0.001), which negatively affects creative cognition (*β* = −0.227, *p* = 0.002). Notably, the model also reveals a significant relationship between self-efficacy and anxiety (*β* = 0.401, *p* < 0.001), suggesting a complex interplay between these mediating factors. This comprehensive model underscores the nuanced effects of AI-integrated design education on creative outcomes, highlighting both the direct benefits and the psychological mechanisms through which it operates.

## 5 Discussion and limitations

### 5.1 Theoretical and practical implications

Our study provides valuable insights into AI’s role in design education and its impact on creative cognition. Using a structural equation model of survey data from 385 design students, we found strong evidence of AI’s positive influence on learners’ creative abilities. Specifically, AI-integrated design education had a significant direct effect on creative cognition (*β* = 0.610, *p* < 0.001) and notable indirect effects mediated through self-efficacy (*β* = 0.232, *p* < 0.001) and anxiety (*β* = −0.084, *p* = 0.003). These results contributed to the growing body of knowledge on technology-enhanced learning, demonstrating the quantifiable benefits of integrating AI into design education.

Building on these findings, our study offered a deeper understanding of the psychological mechanisms through which AI-enhanced learning affects creative cognition. By identifying self-efficacy enhancement and anxiety reduction as crucial mediating factors, our study refines existing theoretical frameworks proposed by Noppe and Gallagher (1977), Runco and Chand (1995), and Beaty et al. (2016).

In the context of existing literature, our findings expanded our understanding of AI’s potential in educational settings, corroborating and extending previous research by Marrone et al. (2022) and Dainys and Jašinauskas (2023). These studies illustrated AI’s capacity to foster creativity and self-confidence among learners. This is particularly significant as it addresses the ongoing debate about AI’s impact on creativity in education, providing evidence contrary to concerns raised by researchers like Solís et al. (2023) regarding AI potentially diminishing students’ creative cognition.

Additionally, our research contributed significantly to the field of educational technology by providing empirical support for and expanding upon the work of Li and Pan (2023). Their research theorized about the potential benefits of AI in educational settings, suggesting that AI systems could enhance students’ innovative thinking skills. Our study moved beyond theoretical propositions by quantifiably demonstrating how AI-integrated design education positively impacted creative cognition through increased self-efficacy and reduced anxiety. Specifically, we found that AI integration not only directly influenced creative cognition but also indirectly enhanced it through psychological mechanisms. This empirical evidence bridged the gap between conceptual frameworks and practical applications, offering a more comprehensive understanding of how AI can be effectively leveraged to foster creativity and innovation in educational contexts.

Looking ahead, these findings opened up exciting avenues for future research and practical applications.

Of broader relevance, the implications of our findings extended far beyond design education. The reduction of anxiety and enhancement of self-efficacy through AI integration could potentially benefit learning across a wide range of subjects and disciplines. For instance, in STEM fields, AI tools could help alleviate anxiety associated with complex problem-solving. Humanities and social sciences could benefit from AI-assisted research tools, empowering students to engage with larger datasets or complex theoretical frameworks. In creative arts and language learning, AI could assist students in overcoming creative blocks and reducing anxiety related to acquiring new skills.

In conclusion, this study contributed to the academic discourse on AI in education and provided a foundation for practical applications that could transform learning experiences across various fields. By understanding the psychological mechanisms through which AI influences creative cognition, we can create educational environments that foster innovation, boost confidence, and prepare students for the complex, technology-driven world they will navigate in their personal and professional lives.

### 5.2 Limitations and directions for future research

Despite the significant findings, this study had several limitations that future research should address. The cross-sectional design limited causal inferences, suggesting a need for longitudinal studies to better understand the relationship between AI use and creative cognition development over time. Our reliance on self-reported surveys may have introduced bias, indicating that future research should incorporate objective measures of creative output and AI proficiency. The study did not explore individual differences based on factors such as gender or academic year, an area that warrants further investigation.

Moreover, the sample was limited to Chinese students, potentially restricting the generalizability of findings. Future research should expand to include students from diverse cultural backgrounds. Additionally, the measures used in this study, while carefully developed, require further validation across different contexts.

Addressing these limitations, future research should focus on deepening the understanding of AI’s impact on design education and creative cognition. Researchers should investigate the long-term effects of AI-integrated education across various disciplines, while educators and policymakers should consider incorporating AI tools into curricula. As the field evolves, exploring the ethical implications of AI in education remains crucial. These collective efforts will contribute to developing effective strategies for AI integration in educational settings, fostering creativity and innovation.

## Data Availability

The original contributions presented in the study are included in the article/supplementary material, further inquiries can be directed to the corresponding author/s.
